# Tissue-Biased and Species-Specific Regulation of Glutathione Peroxidase (*GPx*) Genes in Scallops Exposed to Toxic Dinoflagellates

**DOI:** 10.3390/toxins13010021

**Published:** 2020-12-31

**Authors:** Sein Moh Moh Hlaing, Jiarun Lou, Jie Cheng, Xiaogang Xun, Moli Li, Wei Lu, Xiaoli Hu, Zhenmin Bao

**Affiliations:** 1 Key Laboratory of Marine Genetics and Breeding, College of Marine Life Sciences, Ocean University of China, Ministry of Education, 5 Yushan Road, Qingdao 266003, China; seinmohmohhlaing.myk@gmail.com (S.M.M.H.); ljr@stu.ouc.edu.cn (J.L.); jiecheng@ouc.edu.cn (J.C.); xunxiaogang@qlu.edu.cn (X.X.); lml94520@stu.ouc.edu.cn (M.L.); zmbao@ouc.edu.cn (Z.B.); 2 Laboratory for Marine Fisheries Science and Food Production Processes, Pilot National Laboratory for Marine Science and Technology (Qingdao), 1 Wenhai Road, Qingdao 266237, China

**Keywords:** *Chlamys farreri*, *Patinopecten yessoensis*, glutathione peroxidase (GPx), paralytic shellfish toxin (PST), antioxidant defense, dinoflagellates *Alexandrium*

## Abstract

Marine bivalves could accumulate paralytic shellfish toxins (PSTs) produced by toxic microalgae, which might induce oxidative stress. Glutathione peroxidases (GPxs) are key enzymes functioning in the antioxidant defense, whereas our understanding of their roles in PST challenge in bivalves is limited. Herein, through genome-wide screening, we identified nine (*CfGPx*) and eight (*PyGPx*) *GPx* genes in Zhikong scallop (*Chlamys farreri*) and Yesso scallop (*Patinopecten yessoensis*), respectively, and revealed the expansion of GPx3 sub-family in both species. RNA-Seq analysis revealed high expression of scallop *GPx3*s after D stage larva during early development, and in adult hepatopancreas. However, in scallops exposed to PST-producing dinoflagellates, no *GPx* was significantly induced in the hepatopancreas. In scallop kidneys where PSTs were transformed to higher toxic analogs, most *CfGPx*s were up-regulated, with *CfGPx3*s being acutely and chronically induced by *Alexandrium minutum* and *A*. *catenella* exposure, respectively, but only one *PyGPx* from *GPx3* subfamily was up-regulated by *A*. *catenella* exposure. Our results suggest the function of scallop *GPx*s in protecting kidneys against the oxidative stresses by PST accumulation or transformation. The tissue-, species-, and toxin-dependent expression pattern of scallop *GPx*s also implied their functional diversity in response to toxin exposure.

## 1. Introduction

Reactive oxygen species (ROS) are produced in all living organisms as by-products of aerobic metabolism [1]. The excessive production of ROS can cause lipid peroxidation, protein oxidation, DNA damage, membrane disruption, cellular damage, immune dysfunction, as well as metabolic malfunction [2,3]. Low levels of ROS production are required to maintain the physiological functions, including proliferation, host defense, signal transduction, and gene expression that are involved in the antioxidant defense [4,5]. Aerobic organisms have developed various non-enzymatic and enzymatic antioxidant defense systems to maintain the appropriate level of ROS and protect against the oxidative stress which occurs imbalance between the production of ROS and the antioxidant capacity of the cells [6,7]. Among these enzymatic defense systems, the superoxide dismutase (SOD), catalase (CAT), glutathione S-transferase (GST), and glutathione peroxidase (GPx) are the important enzyme families involved in the detoxification of ROS [6,7]. 

GPxs are important members of the enzymatic antioxidant system that protects organisms from oxidative damage and scavenge peroxides generated in the cells [8]. They catalyze the reduction of H_2_O_2_ and organic hydro-peroxides to water or the corresponding alcohol using glutathione (GSH) as an electron donor [9]. In this way, they maintain a balance between the level of ROS and antioxidants [10]. GPxs are generally classified into two sub-groups: selenium-dependent glutathione peroxidase (SeGPx) and non-selenium glutathione peroxidase (non-SeGPx) based on the presence of selenocysteine (Sec, U) or cysteine (Cys, C) residues at their active sites [11]. The difference between these two enzyme types is that SeGPx catalyzes the reduction of both organic and inorganic peroxides, while non-SeGPx acts only on organic peroxides [12,13,14]. Until now, four major SeGPx isozymes have been reported in mammals [9]: (a) classical GPx (GPx1), (b) gastrointestinal GPx (GPx2), (c) plasma GPx (GPx3), and (d) phospholipid hydroperoxide GPx (PHGPx or GPx4). Moreover, there are also four non-SeGPx isozymes: epididymal GPx (GPx5), olfactory epithelium GPx (GPx6), and two endoplasmic reticulum phospholipid hydroperoxide GPxs (GPx7 and GPx8) [8,11,15,16,17].

GPxs have also been reported in mollusks, which live in diverse aquatic environments and face dynamic stresses. For example, the induction of *GPxs* was observed in response to various bacterial infections, like in Yesso scallop *Patinopecten yessoensis* [18], Zhikong scallop *Chlamys farreri* [19], Manila clam *Venerupis philippinarum* [20], disk abalone *Haliotis discus discus* [21], and clam *Meretrix meretrix* [22], all suggesting the significant role of *GPx*s in innate immune system of mollusks. Moreover, the up-regulation of *GPx*s under different chemical contaminant exposure have also been reported in Manila clam *V. philippinarum* [23], freshwater bivalve *Unio tumidus* [17], mussel *Mytilus galloprovincialis* [16], freshwater bivalve *Anodonta woodiana* [24], thick shell mussel *Mytilus coruscus* [25], Pacific oyster *Crassostrea gigas* [26], and abalone *Haliotis discus hannai* Ino [27], indicating the involvement of mollusk *GPx*s in the oxidative stress response caused by environmental pollutants. 

Bivalves such as mussels, clams, oysters, as well as scallops, are all filter-feeders that can accumulate paralytic shellfish toxins (PSTs) from microalgae, especially the dinoflagellates of the genus *Alexandrium* [28]. PST exposure is known to induce oxidative stress in bivalve species through over-production of ROS [29,30,31]. The activity of antioxidant enzymes, such as SOD, CAT, GST, and GPx, could be enhanced and contributed to the removal of excessive ROS. For example, scallop *SOD* [32] and *GST* [33] genes were both induced after *Alexandrium* exposure; The CAT and GPx enzymes could be oxidative stress biomarkers [34] and were involved in detoxification in responding to PST accumulation in bivalve tissues [35], and the high enzymatic activity also mirrored the gene expression results [36]. Moreover, different organisms may present varied levels of tolerance to PSTs, and the activities of antioxidant enzymes could respond specifically with cell types [37], which indicate that the antioxidant system may be responsible for varied sensitivity to PST exposure. Like many other enzymes, the GPx activities are contributed by the expression of several *GPx* genes. Revealing the expression changes of all the *GPx* members during PSTs exposure could be helpful to our understanding of the antioxidant response variation mediated by GPx in bivalves.

Scallops represent bivalve species in which PSTs could be accumulated at high concentration and with long retaining time [38,39], but systematic analysis of scallop *GPx* genes has not been reported. In this study, we performed whole-genome identification of *GPx* genes in two scallops, Zhikong scallop, *C. farreri* and Yesso scallop, *P. yessoensis*. Their expression profiles in normal scallops and those exposed to PST-producing dinoflagellates were analyzed via RNA-Seq data analysis. We revealed the expansion of scallop *GPx* genes, and their tissue- and species-specific expression pattern during toxic algae challenge. These data expanded our understanding of *GPx* functions in the detoxification and antioxidant defense of shellfish.

## 2. Results and Discussion

### 2.1. Genome-Wide Identification of GPx Genes in C. farreri and P. yessoensis

A total of nine and eight *GPx* genes were identified in *C. farreri* (*CfGPx*) and *P. yessoensis* (*PyGPx*) genomes, respectively. General information regarding their genome position, intron number, encoding protein length, GPx domain position, isoelectric point (pI) value, and molecular weight was summarized in Table 1, and the coding nucleotide and deduced amino acid sequences of *CfGPx*s and *PyGPx*s were shown in Figure S1. In *C. farreri* genome, eight SeGPx genes were identified, including two GPx1s (*CfGPx1-1* and *CfGPx1-2*), five GPx3s (*CfGPx3-1*, *CfGPx3-2*, *CfGPx3-3a*, *CfGPx3-3b*, and *CfGPx3-4*), and one GPx4 (*CfGPx4*), whereas only one non-SeGPx gene, *CfGPx7*, was found. Similarly, in *P. yessoensis* genome, one *PyGPx1*, five GPx3s (*PyGPx3-1*, *PyGPx3-2*, *PyGPx3-3*, *PyGPx3-*4, and *PyGPx3-5*), one *PyGPx4*, and one *PyGPx7* were obtained. Moreover, the coding sequence of *CfGPx*s ranged from 366 to 843 bp in length and encoded proteins from 121 to 280 amino acids (aa), whereas the *PyGPx*s varied from 327 to 834 bp in length and encoded proteins between 108 and 277 aa. The *CfGPx*s had an average theoretical pI from 4.35 to 8.90 and an average molecular weight from 14.22 kDa to 24.68 kDa, while the theoretical pI of *PyGPx*s ranged from 4.43 to 9.41 and the average molecular masses varied from 12.73 kDa to 31.41 kDa. 

Notably, the GPx3 sub-family has more duplicated copies in scallop genomes than in mammal, teleost, and amphibian, indicating the *GPx3* expansion in scallop genome, but members of four GPx sub-families, including GPx2, GPx5, GPx6, and GPx8, were all absent from the scallop genomes (Table 2). Moreover, it was found that the amino acid sequences of *GPx* genes in *C*. *farreri* and *P*. *yessoensis* shared great sequence identity of more than 40% with vertebrates and other mollusks (Table S1), suggesting the conservation of the GPx family between vertebrates and invertebrates. 

### 2.2. Conserved Structures of GPx Genes in C. farreri and P. yessoensis 

**Figure 1:**
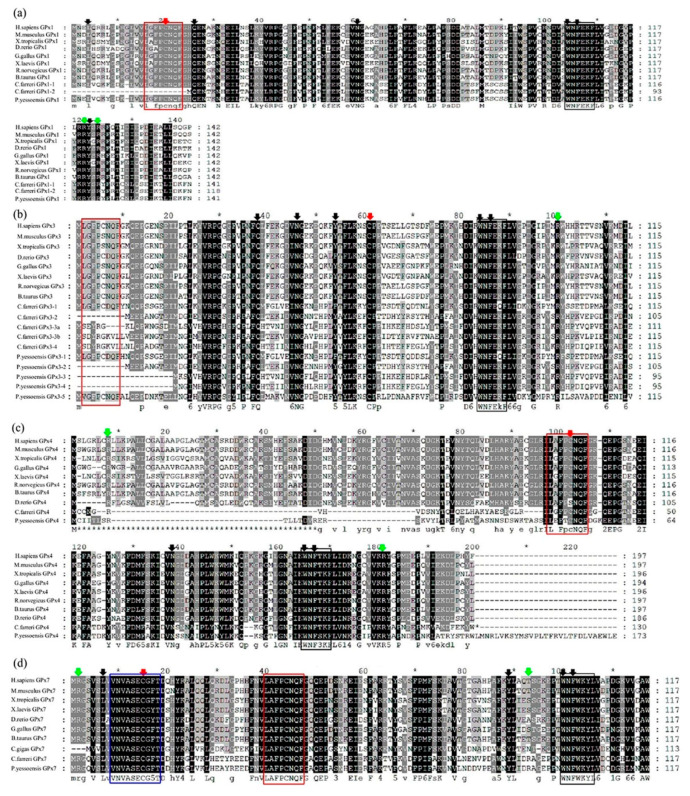
Multiple sequences alignments of the deduced amino acid sequence of *C. farreri* and *P. yessoensis* GPxs: (**a**) GPx1, (**b**) GPx3, (**c**) GPx4, and (**d**) GPx7 with other GPx orthologs deposited in GenBank. The GPx signature motif 1 (blue), GPx signature motif 2 (red) and active site motif (black) were marked by colored frames. The catalytically important residues were indicated by colored down-arrows, as active sites with red, arginine residues with green, and the loop structures with black. Gaps are indicated by dashes to improve the alignments.

The GPx sequence alignment among selected species revealed the presence of conserved amino acid residues, Cys (C), and the active site residues, Gln (Q) and Trp (W) (Figure 1), which were responsible for the fixation of selenium (Sec) [18,19,40]. In addition, the GPx family members were characterized by the presence of a conserved motif (GPx signature 1), (G[K/R]X[L/V][I/L]I[V/E/T]NVA[S/T/A][E/Q/L/Y][C/U]G[L/T]T) with a conserved Cys or Sec residue [41]. Two other conserved GPx domains: GPx signature motif 2, LAFPCNQF and active site motif WNF(S/T)KF, are critical sites for the catalytic activity of this enzyme, which are totally dependent on GSH for its regeneration [41]. It was clear from the sequence alignment that *CfGPx*s and *PyGPx*s shared the conserved features found in well-characterized GPxs from other vertebrates. For example, the typical GPx signature motif 2, L(G/A) (F/V)PC(N/D)QF and the active site motif, WNF(E/T/W)K(F/Y) were also highly conserved in scallop (Figure 1 and Figure S1), although the GPx signature motif 2 was missing in some scallop GPx3 sub-family members. Moreover, the GPx signature motif 1 (GQVSLVVNVASECGFT) was only predicted in the non-SeGPx, GPx7, of both scallops (Figure 1 and Figure S1). Qu et al. have also identified the signature sequence motif, LGFPCNQF, an extra active site motif, WNFEKF, and the GPx-1 active site motif, GKVILVENVASLUGTT, in the N-terminal region of thick shell mussel, *M*. *coruscus* [25]. 

In addition, two arginine (Arg, R) residues which direct the GSH donor substrate towards the catalytic center [42] were also well conserved in both scallop GPxs (Figure 1 and Figure S1). Two arginine residues (R, R) involved in binding GSH, and two amino acid residues glutamine and tryptophan (Q and W) responded for fixation of Sec in GPx were also observed in SeGPx of some mollusks, *M*. *coruscus* [25], *P*. *yessoensis* [18], *C*. *farreri* [19], and *H*. *discus discus* [21]. Moreover, the structural stability of GPx was reported to depend on three-loop structures where the first loop is Asn (N) to Tyr (Y), the second is Leu (L) to Gln (Q), and the third is Trp (W) to Phe (F) [43]. These three loop structures were also conserved in both scallop GPx sequences and were essential for maintaining the biochemical properties and enzymatic activities of GPxs [17,18]. In addition, three other conserved domains and four highly potential catalytic sites (Cys, Gln, Trp, and Asn) were also present in most amino acid sequences of both scallop GPxs (Figure 1 and Figure S1). These conserved amino acids were likely to stabilize GPx structure and function in evolutionary terms.

### 2.3. Phylogenetic Relationship of GPxs between Bivalves and Other Organisms

**Figure 2:**
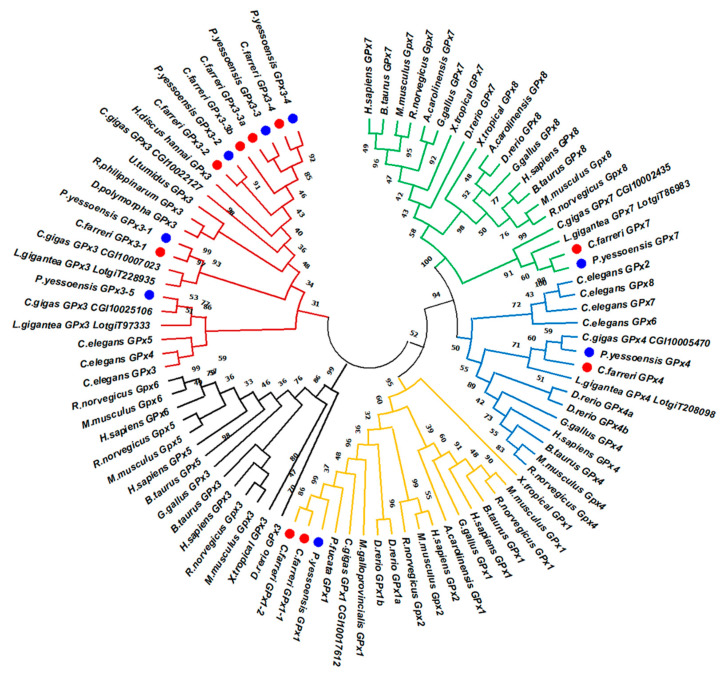
Phylogenetic analysis of GPx family genes of *C. farreri* and *P. yessoensis* with other selected organisms using the maximum-likelihood (ML) method. The numbers next to the branches indicated the bootstrap value of each internal branch in the tree nodes from 1000 replicates. The CfGPxs and PyGPxs were highlighted using red and blue dots, respectively. The colored branches represent GPx1 clade (yellow), mollusk GPx3 sub-clade (red), vertebrate GPx3 sub-clade (black), GPx4 clade (blue), and GPx7/8 clade (green).

Phylogenetic analysis of GPx amino acid sequences from 19 selected species was conducted (Figure 2). As the result, the scallop GPx members can be classified into four major clades. One clade contained vertebrate GPx1 and GPx2 members, as well as mollusk GPx1s clustered with vertebrate GPx2. *CfGPx1*s and *PyGPx*1 formed a sub-cluster with GPx1s from other bivalve species: pearl oyster (*P*. *fucata*), Pacific oyster (*C. gigas*), and Mediterranean mussel (*M*. *galloprovincialis*). In another clade, the GPx3s of both scallops initially formed a marine bivalve GPx3 group and clustered with other mollusk GPx3s, such as Pacific abalone (*H*. *discus hannai*), zebra mussel (*D*. *polymorpha*), freshwater mussel (*U*. *tumidus*), Pacific oyster (*C. gigas*), and Manila clams (*R*. *philippinarum*). All these invertebrate GPx3s formed a sub-clade and clustered with vertebrate GPx3, GPx5, and GPx6 members, which indicated that vertebrate GPx3, 5, and 6 shared the same ancestor with mollusk GPx3 sub-family. Moreover, the GPx4 of both scallops positioned as an independent cluster with a direct linking to the vertebrate GPx4 clade. Besides, the scallop GPx7s were in a clade composed of GPx7s from Pacific oyster, *C*. *gigas* and then clustered with vertebrate GPx7s and GPx8s as the sisters of the well-supported GPx7/8 sub-group, which indicated that the mollusk GPx8 was probably lost in their common ancestor during evolution. The phylogenetic analysis provided evidence that CfGPxs and PyGPxs were derived from a common ancestor with other GPx family proteins as reported by De Zoysa et al. [21]. Zhang et al. also stated that the GPx phylogenetic relationships were in good agreement with traditional taxonomy, suggesting that this protein family might have a primarily similar functional role [20].

### 2.4. Spatio-Temporal Expression of Scallop GPxs During Development and in Adult Tissues

**Figure 3:**
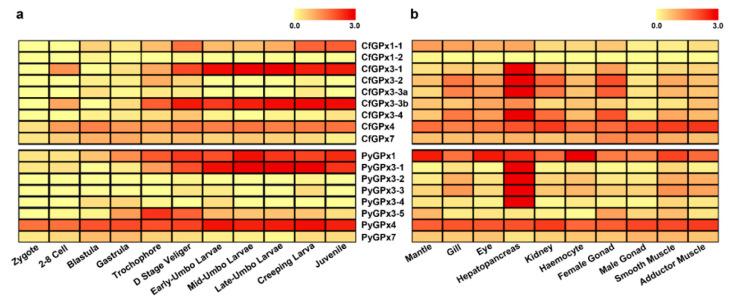
Heatmap of the *GPx* gene expression of *C. farreri* and *P. yessoensis* during embryonic developmental stages (**a**) and in different adult tissues/organs (**b**) with the log_10_(RPKM + 1) values.

The spatio-temporal expression profiles of scallop *GPxs* were analyzed using transcriptome data [38,44]. In scallop embryos and larvae, *CfGPx1-1*, *CfGPx3-1*, *CfGPx3-3b*, and *CfGPx4* in *C. farreri*, and *PyGPx1*, *PyGPx3-1*, *PyGPx3-5*, and *PyGPx4* in *P. yessoensis* exhibited relatively higher expression level than other *GPx*s, suggesting the importance of these *GPx*s in scallop development (Figure 3a). During the entire developmental process, high expression of *GPx* genes (*CfGPx3-1*, *CfGPx3-3b*, *PyGPx1*, *PyGPx3-1*) was mainly present at the larval stages, especially after the D stage veliger, and in the following umbo larvae and juvenile stages (Figure 3a), implying the involvement of these *GPx*s in antioxidation or detoxification in scallop metamorphosis and post-larval development, which may be due to the elevation of oxygen consumption to meet high demands of energy reserve utilization during organ initiation and structural re-modelling [32]. Moreover, both *CfGPx4* and *PyGPx4* were expressed from the very beginning of fertilization and multicellular stages to the larvae and juvenile stages, indicating their maternal origin to play protective roles and to maintain a redox balance during development. 

In adult individuals, the identified scallop *GPx* genes were expressed in all the examined tissues/organs, except that *CfGPx1-2* transcript was not detected in any tissue (Figure 3b). Notably, higher expression of *GPxs* was mainly detected in the hepatopancreas of both scallops. These highly expressed *GPxs* included *CfGPx3-1*, *CfGPx3-2*, *CfGPx3-3a*, *CfGPx3-4*, and *CfGPx4* in *C. farreri*, and *PyGPx1*, *PyGPx3-1*, *PyGPx3-2*, *PyGPx3-3*, *PyGPx3-4*, and *PyGPx4* in *P. yessoensis*, most of which were from the GPx3 sub-family. The dominant *GPx3*s expression in scallop hepatopancreas was consistent with its role as the main metabolic and defense organs against oxidative stress caused by excessive ROS [27], which suggested that scallop *GPx3*s might play crucial roles in molluscan metabolism and antioxidative defense system. The high expression of SeGPx in hepatopancreas was also reported in abalone *H*. *discus discus* [21] and *H*. *discus hannai Ino* [27], freshwater bivalve *U*. *tumidus* [17,45] and *D*. *polymorpha* [40], and clam *M*. *meretrix* [22] and *V*. *philippinarum* [14,20]. In addition, *GPx4*s of both scallops, as well as *PyGPx1*, were transcribed in almost all the examined tissues. Similar expression patterns were also reported in other invertebrates. For example, the *GPx*s of *M. galloprovincialis* were mainly expressed in the gills, hemocytes, and digestive gland, moderately expressed in adductor muscle and gonad [16], and the *GPx* of *L*. *vannamei* was widely distributed in the hemocytes, hepatopancreas, gills, muscles, and intestine tissues [46].

### 2.5. Expression Regulation of Scallop GPxs in Response to PST-Producing Dinoflagellates

As filter-feeders, scallops have a high ability to accumulate PSTs [37]. Previous studies have suggested that the scallop hepatopancreas and kidneys were the two major organs involved in PST bioaccumulation and transformation, respectively [38,47,48]. Moreover, dinoflagellates of the genus *Alexandrium* are the major PST producers containing varied toxic analogs among species and strains. Therefore, to understand the scallop defense mechanism against toxic algae, RNA-Seq analysis was performed to determine the expression profiles of *GPx* genes in scallop kidneys and hepatopancreas in response to the ingestion of PST-producing algae, *A*. *minutum* (AM-1 strain) and *A*. *catenella* (ACDH strain) (Tables S2 and S3). 

**Figure 4:**
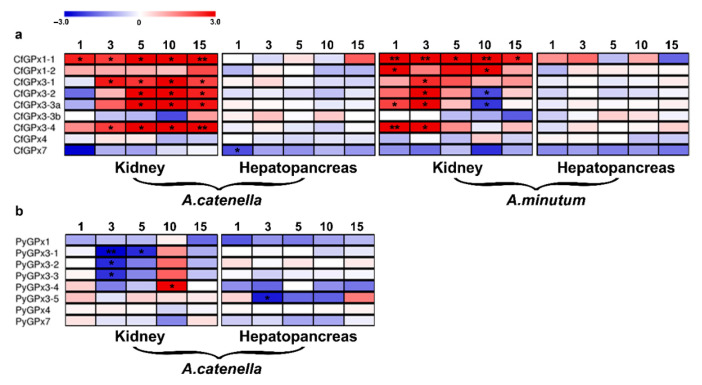
Temporal expression of scallop *GPx*s in hepatopancreas and kidneys of *C. farreri* after *A. minutum* and *A. catenella* exposure (**a**), and of *P. yessoensis* after *A. catenella* exposure (**b**). The heatmaps were drawn based on log_2_FC of the exposed group to the control group (0 day) (Table S3). Exposure time (1, 3, 5, 10, or 15 days) was displayed above each heatmap. Significant regulation (|log_2_FC| > 2 and *p* < 0.05) was indicated with “*”, and very significant regulation (|log_2_FC| > 2 and FDR < 0.05) was indicated with “**”.

In *C. farreri* hepatopancreas, the major organ for PST accumulation from algae [38,48], most *CfGPx*s’ expression was not significantly altered after toxic algae exposure, with only *CfGPx7* down-regulated at the 1st day after *A. catenella* exposure (log_2_FC: −2.06) (Figure 4a). This result implied that *CfGPx*s mediated antioxidant defense in hepatopancreas was not sensitive to PST challenge, and the highly expressed *CfGPx3*s might provide stable antioxidant capacity for redox homeostasis during toxic algae ingestion. 

In *C. farreri* kidneys, where the ingested PSTs could be transformed to higher toxic analogs [38], a prominent up-regulation of *CfGPx1-1*, *CfGPx3-1*, *CfGPx3-2*, *CfGPx3-3a*, and *CfGPx3-4* was detected after *A*. *catenella* exposure (log_2_FC: 2.18–5.61) (Figure 4a). Similarly, *A*. *minutum* exposure significantly induced the up-regulation of *CfGPx1-1, CfGPx1-2, CfGPx3-1, CfGPx3-2, CfGPx3-3a,* and *CfGPx3-4* in this organ (log_2_FC: 2.19–5.88) (Figure 4a). For both toxic algae challenge, the majority of up-regulated *CfGPx*s in kidney were from the GPx1 and GPx3 sub-families, suggesting the importance of GPx1 and GPx3 members in PST detoxification in scallop kidneys, especially during the transformation of PSTs to more potent analogs [38]. The duplication of *CfGPx3*s and *CfGPx1*s might be related to the detoxification of *C. farreri* with respect to PST metabolism in the toxin transformation center kidney. 

However, the regulation of *CfGPx*s in scallop kidneys exhibited different patterns between the two toxic algae exposure (Figure 4a). For example, the induction of *CfGPx3*s was mostly chronic (5th–10th days, log_2_FC: 2.18–5.61) with *A. catenella* challenge, whereas acute (1st–3rd days, log_2_FC: 2.19–5.45) with *A. minutum* exposure. Moreover, the induction of *CfGPx1-2* and the down-regulation of *CfGP3x*s were only observed in the kidney of scallops exposed to *A. minutum* (Figure 4a and Table S3). Since the two micro-algae produce different PST analogs, with *A. minutum* mainly synthesizing gonyautoxins (GTXs, mainly GTX1-4) and *A. catenella* synthesizing *N*-sulfocarbamoyl toxins (C1 and C2) [38,48], it therefore suggested that the difference in time dependent pattern of *GPx3*s response between *A. catenella* and *A. minutum* challenge in *C. farreri* kidneys was probably due to the different toxin species accumulated (Figure S2). This diverse expression profiles of *CfGPx*s indicated that, with the challenges of different PST analogs, the *CfGPx*s functioning in PST detoxification and antioxidation during PST transformation were similar in scallop kidneys, but the gene stimulation or inhibition procedure/speed might vary.

To further compare the regulation of *GPx*s between scallop species, we examined *PyGPx*s transcripts in both hepatopancreas and kidneys of *P. yessoensis* ingesting *A. catenella* (Figure 4b). There were less regulated *GPx*s than those identified in *C. farreri*, with only *PyGPx3-4* being up-regulated in *P. yessoensis* kidney (10th day, log_2_FC: 2.88) and three *PyGPx3*s down-regulated (3rd and 5th days, log_2_FC: −2.42–−2.95) (Figure 4b). In *P. yessoensis* hepatopancreas, only *PyGPx3-5* was down-regulated at the 3rd day (log_2_FC: −2.60). The inhibition of *PyGPx*s might be resulted from the decline of GSH contents under cellular redox imbalance, to maintain the stable GSH/GSSG ratio which is crucial in preventing oxidative damage [49]. Moreover, the species-specific and tissue-biased expression of genes from the expanded GPx3 sub-family may also indicate the different sensitivity of scallops to varied PSTs (Figure S2) and the possible association of GPx3 expansion in the adaptation of scallops to the environment with different toxic algae. 

The antioxidant metabolism is crucial for preventing the cellular oxidative damage in bivalves exposed to toxic dinoflagellates, such as *Alexandrium* sp. [30]. Stress response is usually modulated at the molecular level and shows significant changes in the transcripts expressed in the target tissues [50]. Many studies have described the impact of PST-producers on gene expression of diverse bivalve organs, majorly in *C. gigas*. For example, both repression and activation of genes in *C*. *gigas* gills by *A*. *minutum* induction was involved in key metabolic pathways such as oxidative stress, mitochondrial metabolism, endogenous clock, immunity, and detoxification processes, including *SODs, GSTs,* and *GPxs* [30,51]. The expression of defense-related genes modulated in the hemocytes of oyster after exposure to *A*. *minutum* could demonstrate the impact of direct contacting of some *Alexandrium* species on different cell types due to the production of extracellular compounds [52]. Expression of genes involved in antioxidant stress, detoxification, immune, and stress defense of *C*. *gigas* juveniles increased in a short exposure time by the toxic dinoflagellate, *G*. *catenatum* [53,54] and *P. lima* exposure [55]. Therefore, the high expression level of *CfGP3x*s maintained in scallop hepatopancreas, as well as the significantly regulated expression of *CfGPx*s in kidneys reasonably supported the fact that these two organs were the main centers involved in detoxification and antioxidant defense, but with different roles. These findings also indicated that the expression of *GPx* genes varied among scallop species and tissues, dinoflagellate strains, as well as the exposure time. The species- and organ-specific regulation of *GPx*s imply the diverse function of scallop *GPx*s in the defense against the harmful effects of PST accumulation and metabolism, which might contribute to the scallop adaptation to diverse marine environments. 

## 3. Conclusions

In conclusion, a total of nine *CfGPx* and eight *PyGPx* genes were identified in scallops *C. farreri* and *P. yessoensis*, respectively, with the expansion of GPx3 sub-family in both species. *GPx3*s showed higher expression than other *GPx* genes, and hepatopancreas was the major organ for scallop *GPx* expression. After ingesting PST-producing algae, no *GPx* was induced in hepatopancreas of either scallop, but most *GPx*s were up-regulated in *C. farreri* kidneys, with acute and chronic regulation by *A*. *minutum* and *A. catenella* exposure, respectively. Meanwhile, only one *GPx3* was induced in kidneys of *P. yessoensis* exposed to *A. catenella*. These results indicated that the response of scallop *GPx*s to PST-producing dinoflagellates was dependent on scallop species and tissues, as well as dinoflagellates strains. Our data revealed the involvement of *GPx*s in scallops against the toxic stress and highlighted their functional importance, which provided a better understanding of the detoxification systems in scallops and their regulation mechanism.

## 4. Materials and Methods

### 4.1. Screening GPx Genes from the C. farreri and P. yessoensis Genomes

The *GPx* genes were identified using the *C. farreri* and *P. yessoensis* transcriptome and genome data [38,44]. The coding sequences were translated using the ORF finder (https://www.ncbi.nlm.nih.gov/orffinder/), and the predicted ORFs were validated using the NCBI non-redundant (Nr) protein sequence database. The protein sequences of *GPxs* were further verified with BLASTP in NCBI (http://blast.ncbi.nlm.nih.gov/) and UniProt (http://www.uniprot.org/) with the setting of e-value to 1E-05. All possible GPx proteins from the BLASTP results were then tested using SMART (http://smart.embl-heidelberg.de/). Protein sequences longer than 100 amino acids were used for further analysis. The translated sequences were submitted to the SMART program for identification of the signal peptide and other conserved GPx structures. Compute pI/MW tool (https://www.expasy.org/) was used to predict the pI value and molecular weight (kDa). 

### 4.2. Multiple Sequence Alignment and Phylogenetic Analysis of the GPx Gene Family

Multiple sequence alignment of GPx sequences was performed using Clustal W [56] and then was edited by Genedoc software (version 2.7.0) [57]. The amino acid similarity matrix was made using the Clustal Omega software (http://www.ebi.ac.uk/Tools/msa/clustalo/) with GPx protein sequences of both scallops and other selected species. The GPx amino acid sequences of selected species included *Homo sapiens*, *Mus musculus*, *Bos taurus*, *Rattus norvegicus*, *Xenopus tropicalis*, *Anolis carolinensis*, *Gallus gallus, Danio rerio*, and mollusk species such as *Pinctada fucata*, *U. tumidus*, *H. discus hannai*, *Dreissena polymorpha*, *Ruditapes philippinarum*, *C. gigas*, and *M. galloprovincialis*, all obtained from Ensembl (http://www.ensembl.org/) and NCBI (https://www.ncbi.nlm.nih.gov/) (Table S4). The phylogenetic tree was constructed using the GPx amino acid sequences of *C*. *farreri* and *P*. *yessoensis*, as well as other selected species using MEGA 7.0 with the maximum-likelihood (ML) method [58] and the tested best model of LG + G. The bootstrap analysis with 1000 replicates was applied to test the relative support for the branches produced by ML method. 

### 4.3. Expression Analysis of GPxs During Scallop Development and in Adult Organs/Tissues

*C. farreri* and *P. yessoensis* were collected from Xunshan Group Co., Ltd. (Rongcheng, Shandong Province, China) and Zhangzidao Group Co., Ltd. (Dalian, Liaoning Province, China), respectively. The RNA-Seq data for scallop embryo/larva including zygote, 2–8 cells, blastula, gastrula, trochophore, D stage larvae, early umbo larvae, mid umbo larvae, late umbo larvae, creeping larvae, and juvenile scallops, and the adult organs/tissues including mantle, gill, eye, kidney, hepatopancreas, hemocyte, female gonad, male gonad, smooth muscle, and adductor muscle, were analyzed. The expression profiles of *CfGPx*s and *PyGPx*s during development and in adult organs/tissues were normalized and represented in the form of RPKM (Reads Per Kilobase of exon model per Million mapped reads) using the data from our previous studies [38,44]. To visualize the expression patterns of *GPx* genes on both scallops, the heatmap was generated via heatmap package under the R software environment with the log_10_(RPKM + 1) value.

### 4.4. Expression Analysis of GPx Genes in Scallops Exposed to Toxic Dinoflagellates

The scallops *C. farreri* were exposed to PST-producing dinoflagellates, *A*. *minutum* (AM-1 strain) and *A*. *catenella* (ACDH strain) [38,48]. Briefly, 2-year-old adult scallops were collected and acclimated in filtered and aerated seawater at 12–13 °C for three weeks for depuration by feeding the non-toxic algae, *Isochrysis galbana* (7.5 × 10^5^ cells/mL) as the control group [59]. 18 scallops were separated into independent tanks with aeration, and 6 groups with 3 individuals for each were taken at different test time points (0, 1, 3, 5, 10, and 15 days) during the challenging experiments. The two *Alexandrium* dinoflagellate strains were cultured independently in F/2 medium using artificial seawater, and were harvested at the late exponential growth phase through centrifugation of 2500 g/10 min [54,60]. Then, each scallop was fed once a day with 3 L (2.5 × 10^3^ cells/mL) *A*. *minutum* or *A*. *catenella*. For comparison, 2-year-old *P. yessoensis* were also exposed to *A*. *catenella* (ACDH strain) using the same procedure described above. Scallops were sampled at 0 day (control), 1 and 3 (acute response) days, 5, 10, and 15 (sub-chronic reaction) days with three individuals at each time point after AM-1 and ACDH exposure. The kidney and hepatopancreas of these scallops were dissected, washed with sterile seawater, and immediately frozen at −80 °C for subsequent RNA extraction. PST concentrations in the tissues of sampled scallops were determined by high-performance liquid chromatography with tandem mass spectrometry (HPLC-MS/MS) method [38].

Total RNA was isolated from scallop kidneys and hepatopancreas, and the RNA quantity and quality were checked by Nanovue Plus and gel electrophoresis before the following reverse transcription and sequencing analysis. RNA-Seq data obtained from these tissues were used to examine the expression profiles of *GPx* genes in response to different toxic dinoflagellates. Briefly, the RNA-Seq libraries of these tissues from *C*. *farreri* and *P*. *yessoensis* fed with AM-1 and ACDH were constructed independently using the NEB Next mRNA Library Prep Kit following the manufacturer’s instructions and were subjected to PE125 sequencing on the Illumina HiSeq 2000 platform. The RNA-Seq reads were mapped to the *C*. *farreri* and *P*. *yessoensis* genomes, respectively, using Tophat (ver 2.0.9), and the expression of all *GPx* genes were normalized and represented in the form of RPKM (Table S2). The fold change (FC) between the toxin-exposed and control groups was calculated and the significant differences were determined using the edgeR package with cutoff of |log_2_FC| > 2 and *p* value < 0.05, and the very significant difference with cutoff of |log_2_FC| > 2 and corrected FDR value < 0.05. The heat maps were drawn using pheatmap package with the log_2_FC values (Table S3).

## Figures and Tables

**Table 1:** General information of the *GPx* genes in *C. farreri* and *P. yessoensis* genomes.

Gene	Genomic Position	No. Intron	Amino Acid (aa)	GPx Domain Position	Isoelectric Point (pI)	Molecular Weight (KDa)
*CfGPx1-1*	20345.14:285273–290196	1	145	1–71	7.63	16.92
*CfGPx1-2*	723835.1:769–1754	1	121	1–47	7.66	14.22
*CfGPx3-1*	16535.52:1096572–1102808	2	130	1–63	5.86	16.33
*CfGPx3-2*	41509.77:1599940–1606070	2	145	1–73	5.62	16.23
*CfGPx3-3a*	41509.75:1596971–1598762	2	173	1–24	6.70	19.79
*CfGPx3-3b*	41509.78:1608479–1610027	2	221	96–143	8.24	24.68
*CfGPx3-4*	41509.74:1587475–1591661	2	192	1–24	6.23	22.01
*CfGPx4*	57527.11:265310–274184	4	141	3–83	8.90	15.00
*CfGPx7*	61639.7:153594–157975	3	280	102–210	4.35	31.83
*PyGPx1*	7781.4:46294–55646	1	143	1–71	8.73	16.67
*PyGPx3-1*	2921.10:304644–315700	2	141	1–63	5.03	16.09
*PyGPx3-2*	7441.4:20641–24946	2	141	1–73	5.40	15.89
*PyGPx3-3*	7441.1:18051–19124	1	108	1–94	9.41	12.73
*PyGPx3-4*	9581.1:21483–23254	1	108	1–100	8.76	12.83
*PyGPx3-5*	10997.2:56916–60988	2	203	3–108	8.31	23.38
*PyGPx4*	2091.24:1003767–1022307	4	173	9–97	9.26	20.10
*PyGPx7*	7907.21:716289–720446	3	277	99–207	4.43	31.41

**Table 2:** Copy number of *GPx* genes in vertebrates and mollusk genomes.

*GPx*	*H. sapiens*	*M. musculus*	*A. carolinensis*	*G. gallus*	*X. tropicalis*	*D. rerio*	*L. gigantea*	*C. gigas*	*C. farreri*	*P. yessoensis*
*GPx1*	1	1	1	1	1	2	0	1	2	1
*GPx2*	1	1	0	0	0	0	0	0	0	0
*GPx3*	1	1	0	1	1	1	2	3	5	5
*GPx4*	1	1	0	1	0	2	1	1	1	1
*GPx5*	1	1	0	0	0	0	0	0	0	0
*GPx6*	1	1	0	0	0	0	0	0	0	0
*GPx7*	1	1	1	1	1	1	1	1	1	1
*GPx8*	1	1	1	1	1	1	0	0	0	0
Total	8	8	3	5	4	7	4	6	9	8

## Data Availability

The RNA-Seq data for exposure experiments in this study were deposited in NCBI Sequence Read Archive, with the accession numbers of SRX2445405-SRX2445440, and the RPKM values and differential expression results of *GPx*s were in supplementary Tables S2 and S3.

## Supplementary Materials

The following are available online at https://www.mdpi.com/2072-6651/13/1/21/s1, Table S1. The homology percentages of CfGPx and PyGPx amino acid sequences identity compared with other animals, Table S2. RPKM values of *GPx*s from RNA-Seq analysis after toxic *A. catenella* and *A. miutum* exposure in hepatopancreas and kidneys at different time points, Table S3. Different expression of *GPxs* in hepatopancreas and kidneys after *A. catenella* and *A. minutum* exposure analyzed using edgeR package, Table S4. Information of *GPx*s used in this study from vertebrates and mollusk species, Figure S1. Nucleotide and deduced amino acid sequences of (1) CfGPxs and (2) PyGPxs, Figure S2. The temporal abundance of PSTs in scallop kidneys and hepatopancreas after exposure to the toxic alga *A. minutum* and *A. catenella*.
