# Supplementary material for: Tissue-Biased and Species-Specific Regulation of Glutathione Peroxidase (GPx) Genes in Scallops Exposed to Toxic Dinoflagellates
Source: Toxins (Basel). 2020 Dec 31;13(1):21. doi: 10.3390/toxins13010021 (PMC7824116; doi:10.3390/toxins13010021)
Supplement: Supplementary file 1 [file toxins-13-00021-s001.zip › Supplementary Files/Figure S1-S2.docx]

Supplementary Materials: Tissue-Biased and Species-Specific Regulation of Glutathione Peroxidase (*GPx*) Genes in Scallops Exposed to Toxic Dinoflagellates

Sein Mohmoh Hlaing, Jiarun Lou, Jie Cheng, Xiaogang Xun, Moli Li, Wei Lu, Xiaoli Hu and Zhenmin Bao


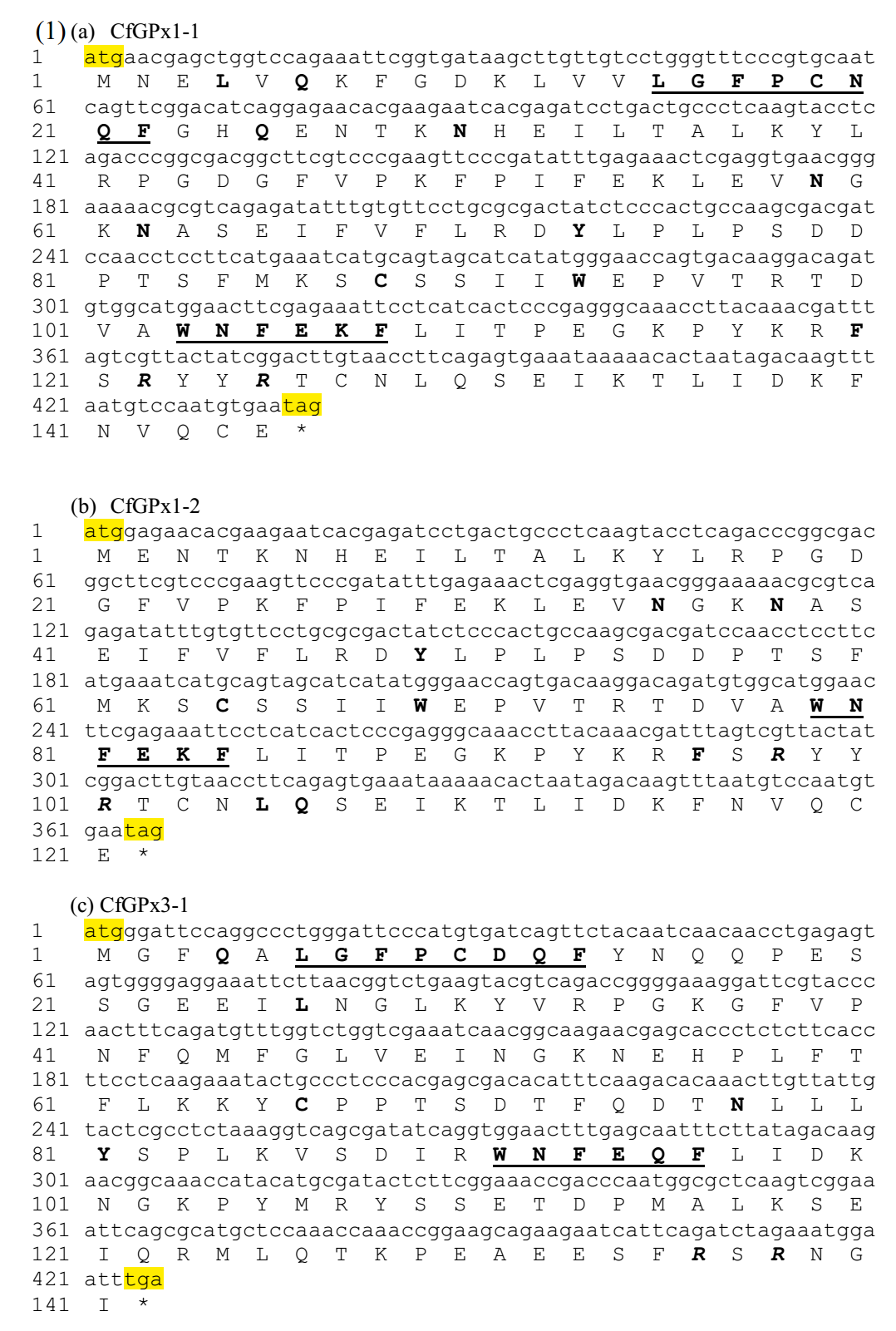


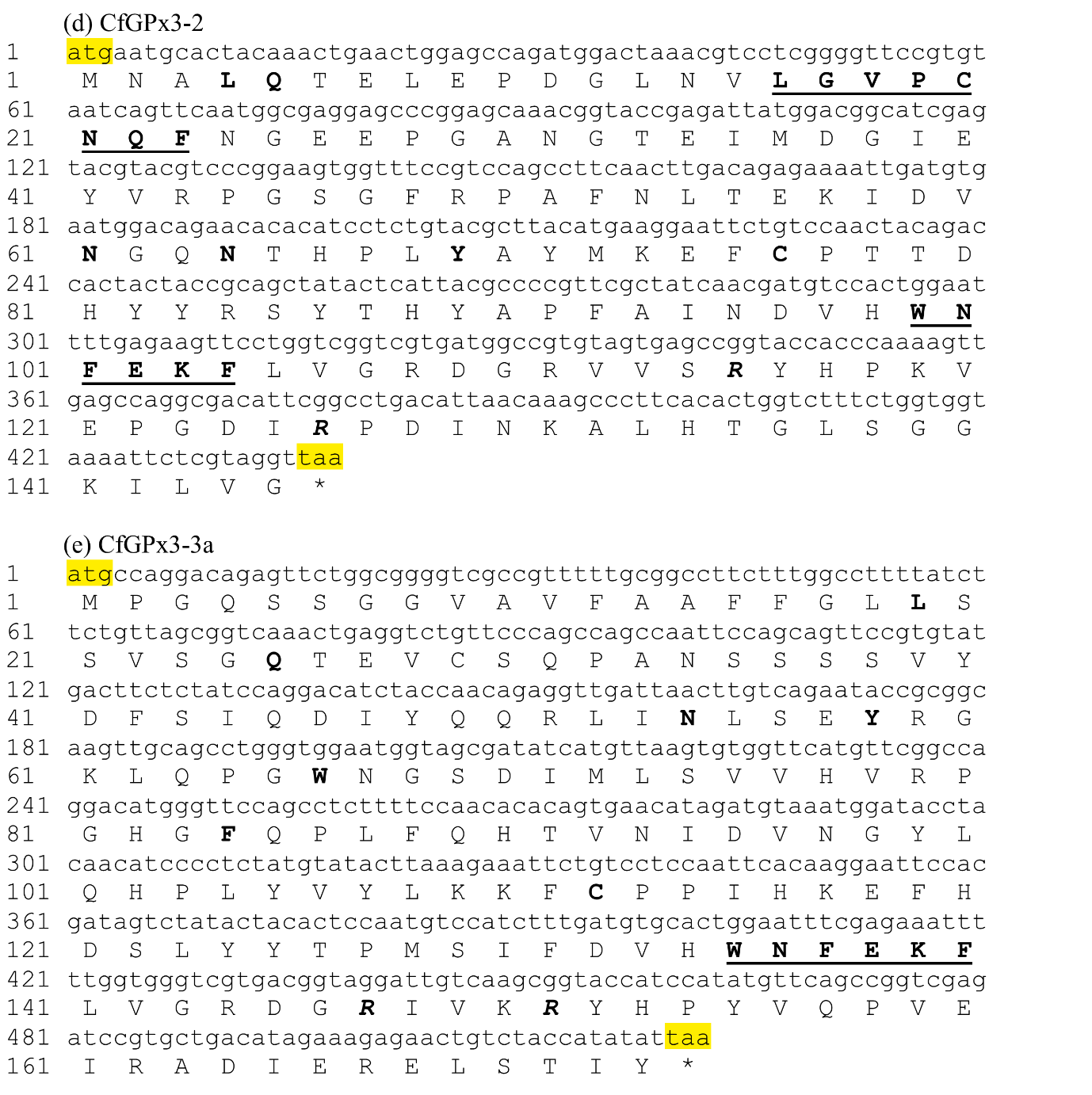

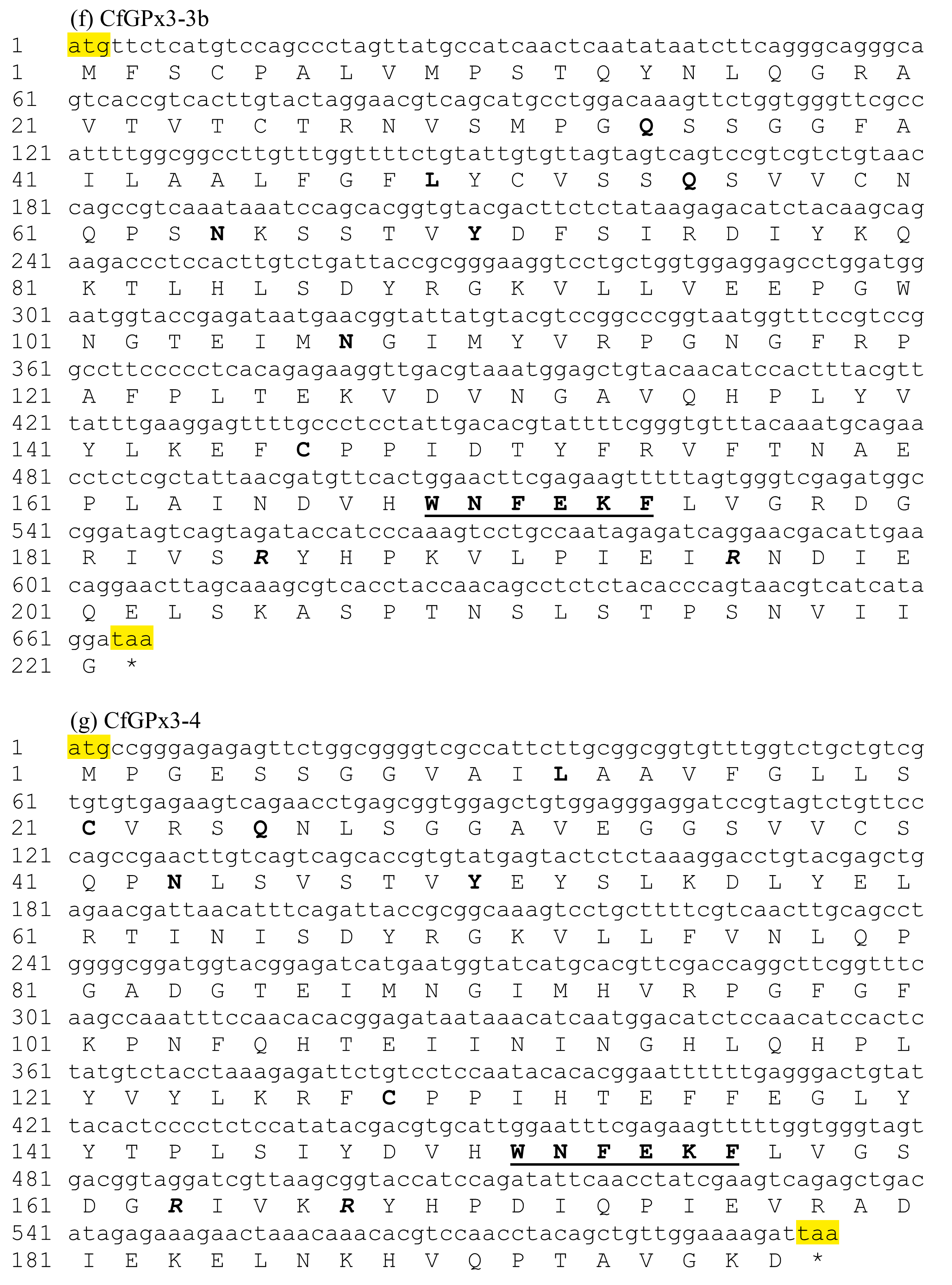

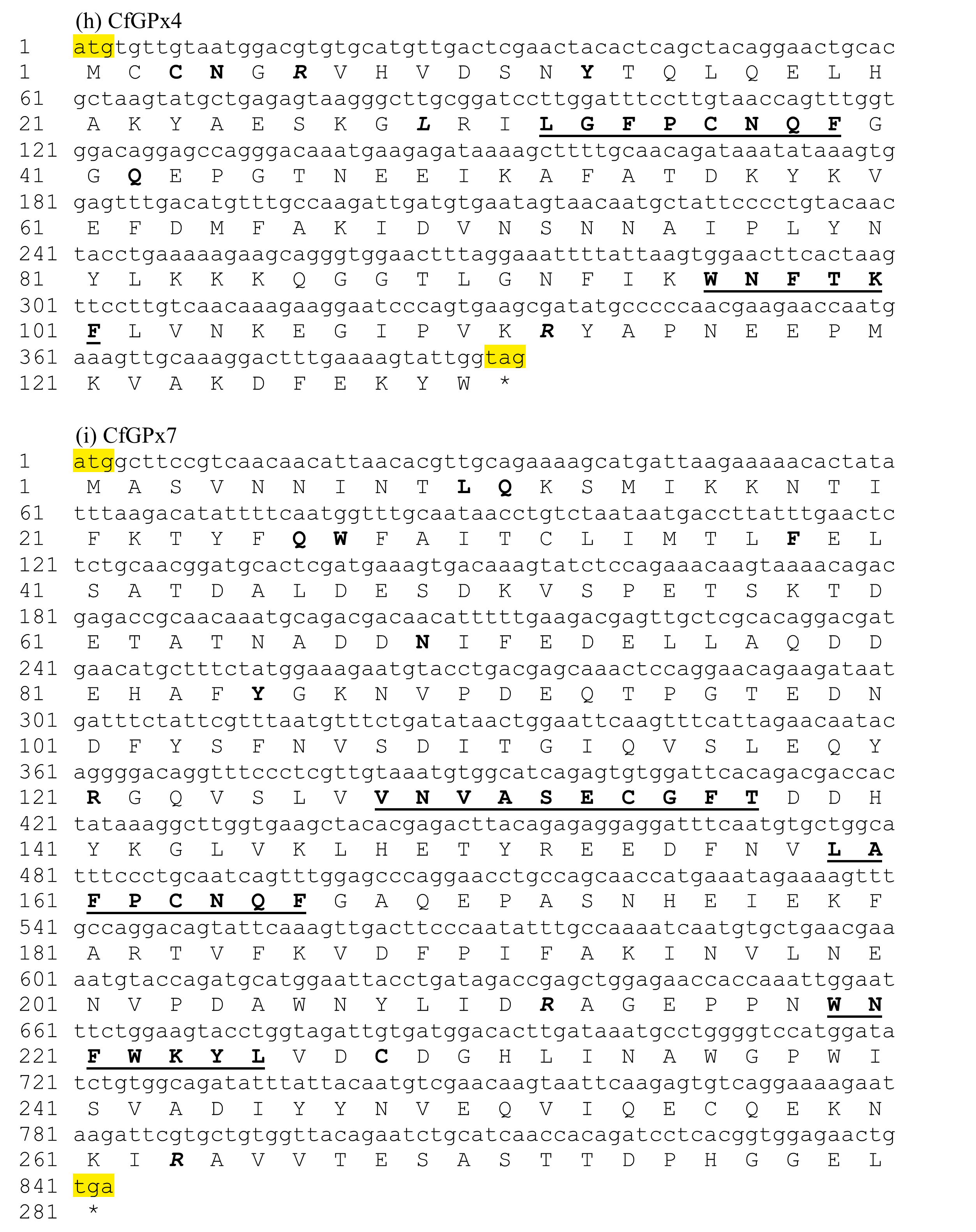

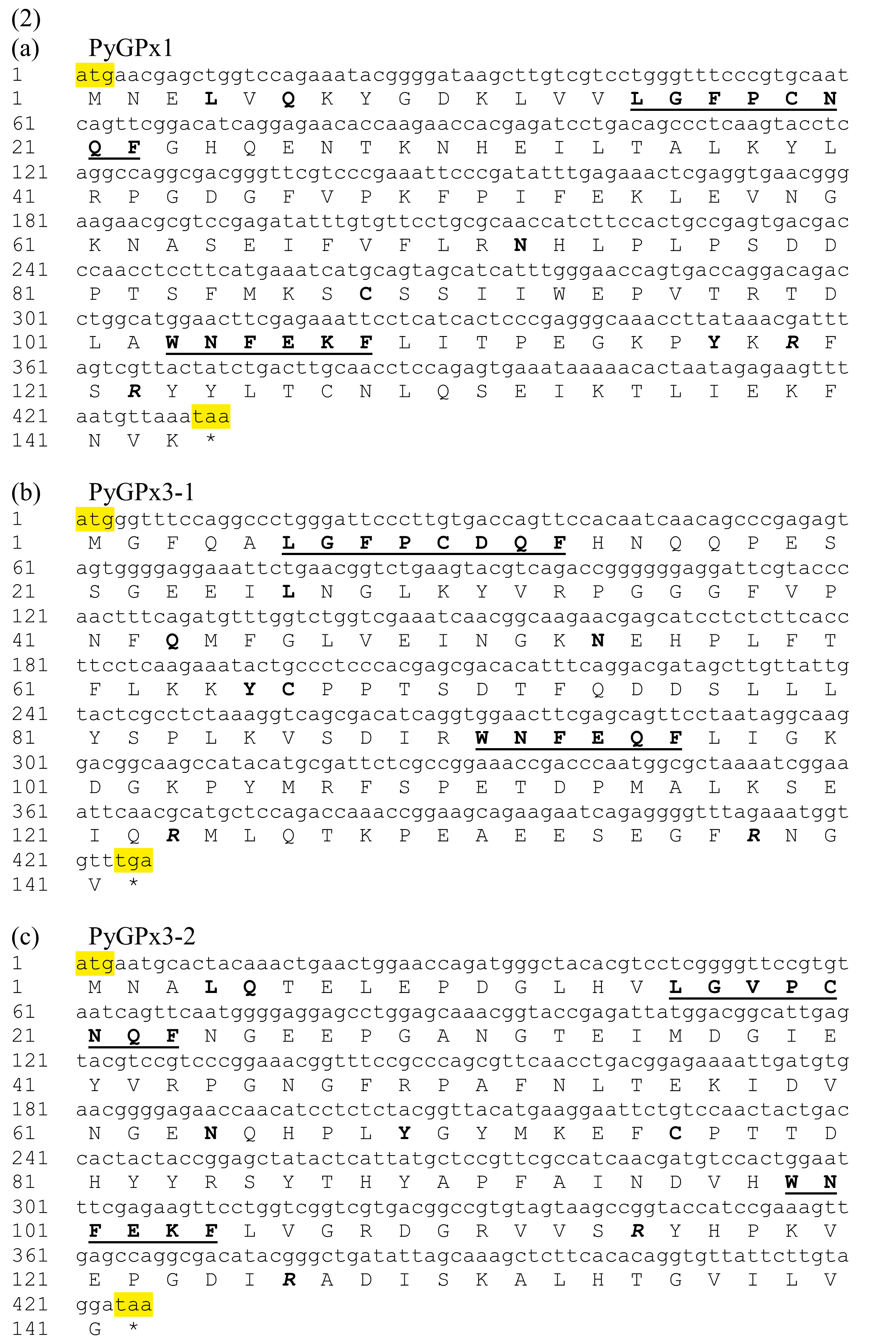

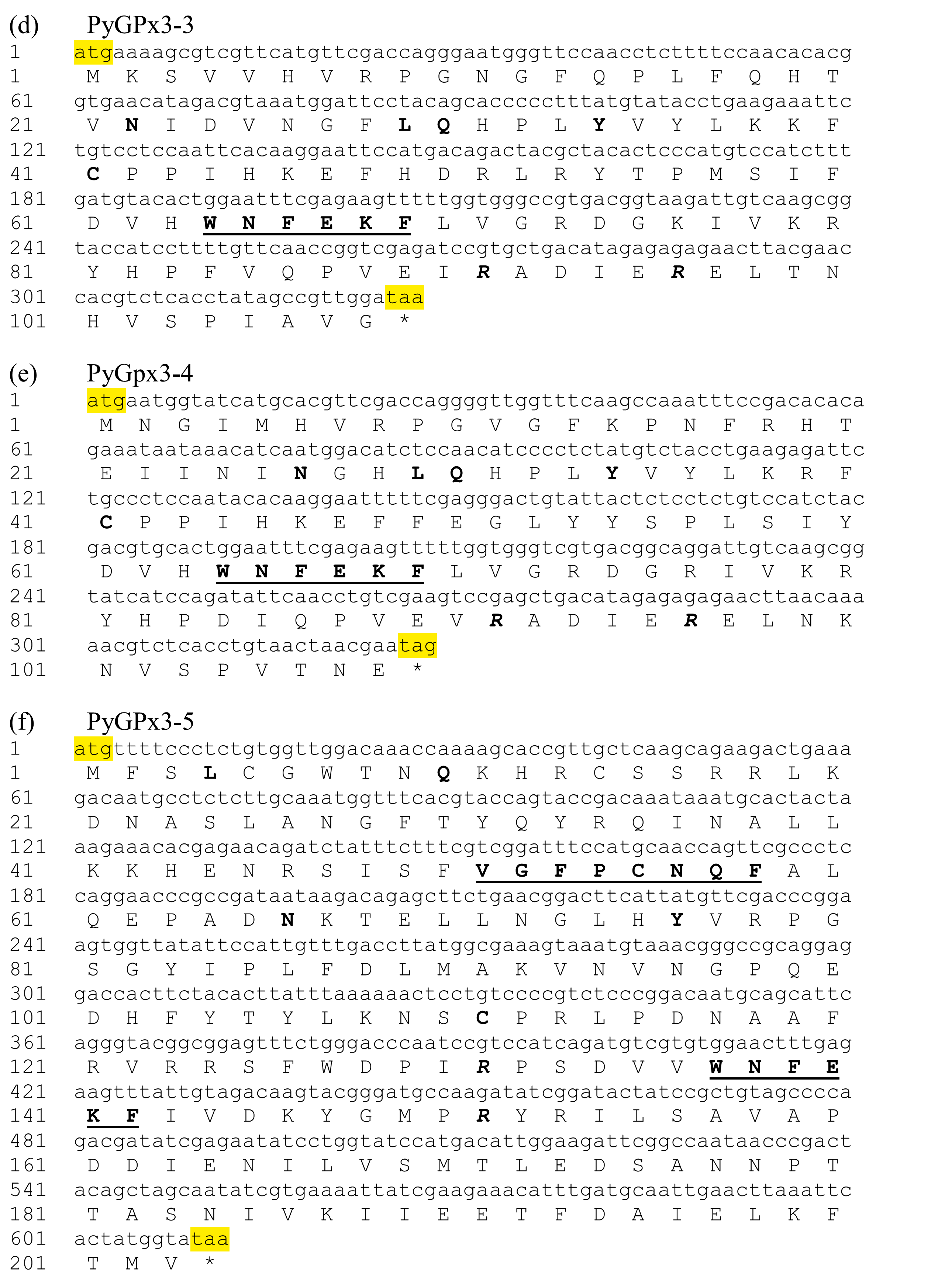

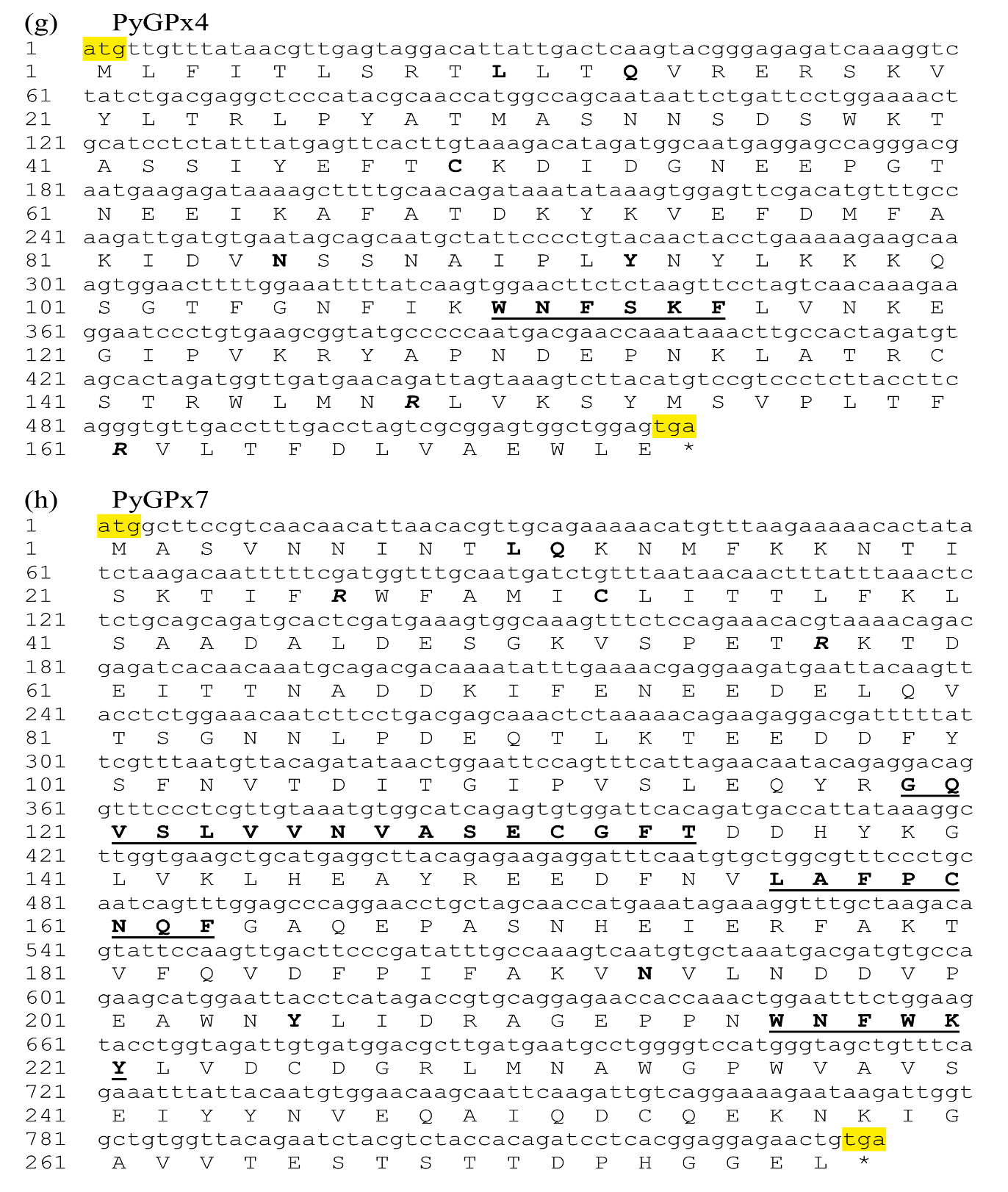


**Figure S1.** Nucleotide and deduced amino acid sequences of (1) CfGPxs and (2) PyGPxs. The nucleotides and amino acids are numbered along the right and left margin, respectively. The start (atg) and stop (tag, tga, and taa) codons are highlighted with yellow color. The asterisk (*) indicates the stop codon. The Catalytically important amino acid residues, glu (Q), try (W) and cys (C), involved in binding glutathione are highlighted with bold. The GPx signature motif 1 (GQVSLVVNVASECGFT), the GPx signature motif 2 ((V/L)(G/F)VPCNQF) and the GPx active site motif (W(N/T)F(E/W) KF) are bolded and underlined. The potential N-glycosylation residues are highlighted with black shadow. Two arginine (R, R) residues for directing donor glutathione substrate are italicized and bolded. The structural stability of GPx depends on three-loop structures, Asn (N) to Tyr (Y), Leu (L) to Gln (Q) and Trp (W) to Phe (F) are bolded.


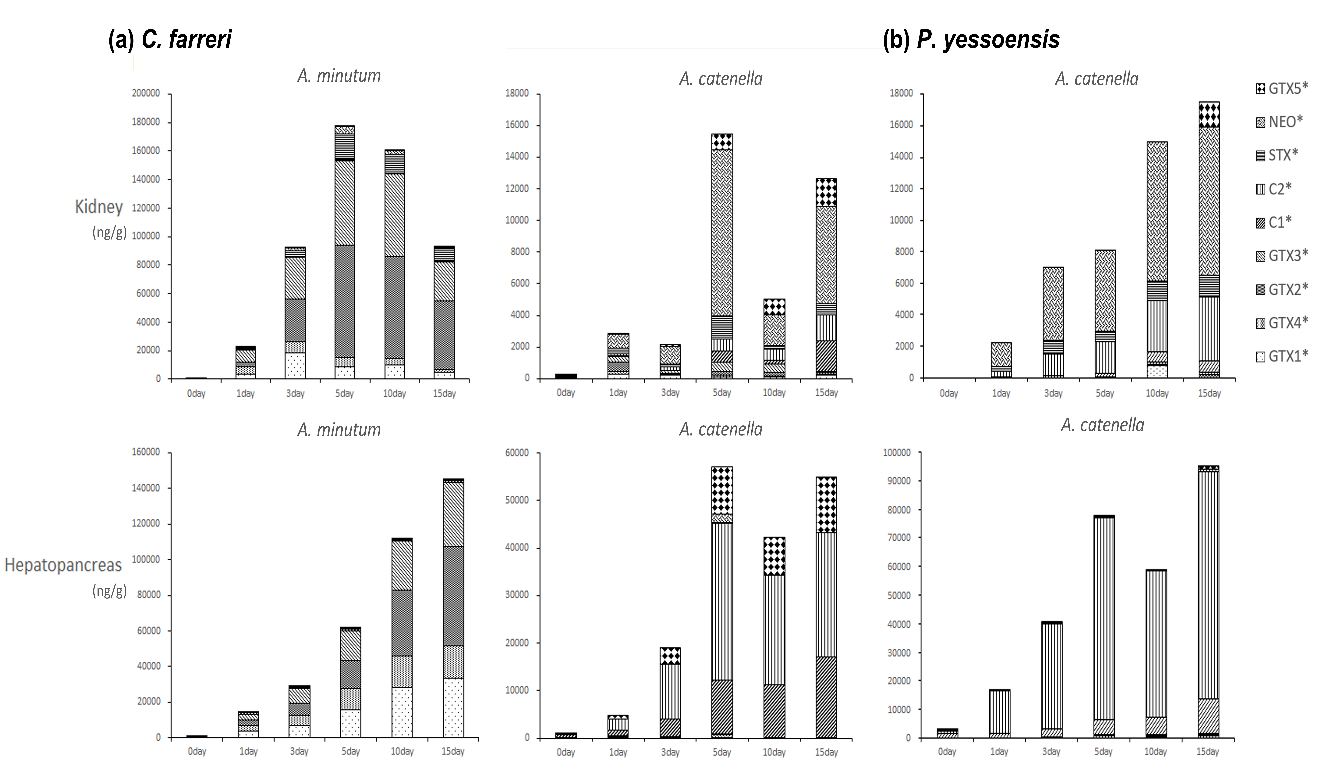
**Figure S2.** The temporal abundance of PSTs in scallop kidneys and hepatopancreas after exposure to the toxic alga *A. minutum* and *A. catenella*.
