# Supplementary material for: Tissue-Biased and Species-Specific Regulation of Glutathione Peroxidase (GPx) Genes in Scallops Exposed to Toxic Dinoflagellates
Source: Toxins (Basel). 2020 Dec 31;13(1):21. doi: 10.3390/toxins13010021 (PMC7824116; doi:10.3390/toxins13010021)
Supplement: Supplementary file 1 [file toxins-13-00021-s001.zip › Supplementary Files/Table S4-GPx Seq Similarity.docx]

Supplementary Materials: Tissue-Biased and Species-Specific Regulation of Glutathione Peroxidase (*GPx*) Genes in Scallops Exposed to Toxic Dinoflagellates

Sein Mohmoh Hlaing, Jiarun Lou, Jie Cheng, Xiaogang Xun, Moli Li, Wei Lu, Xiaoli Hu and Zhenmin Bao

**Table S4.** The homology percentages of CfGPx and PyGPx amino acid sequences identity compared with other animals.

| **Gene** | **Vertebrates** | | | | | | | | **Mollusks** | | | | | | |
| --- | --- | --- | --- | --- | --- | --- | --- | --- | --- | --- | --- | --- | --- | --- | --- |
|  | **Hs** | **Mm** | **Xt** | **Dr** | **Gg** | **Xl** | **Rn** | **Bt** | **Pf** | **Ut** | **Hdh** | **Dp** | **Rp** | **Cg** | **Mg** |
| *CfGPx1-1* | 73.1 | 58.6 | 55 | 60 | 57.1 | 55.7 | 57.9 | 56.4 | 66 | 39.3 | 32.4 | 37.1 | 38.6 | - | 71.8 |
| *CfGPx1-2* | 53.5 | 55.7 | 54.4 | 58 | 54.4 | 53.5 | 55.3 | 53.5 | 62.7 | 38.9 | 30.6 | 34.6 | 38.3 | - | 68.4 |
| *CfGPx3-1* | 50 | 47.5 | 44.7 | 50.8 | 45 | 47.5 | 48.33 | 45.8 | 40.3 | 48.1 | 43.1 | 44.3 | 47 | - | 44.8 |
| *CfGPx3-2* | 45.8 | 46.6 | 46.2 | 42.4 | 44.3 | 44.7 | 46.6 | 46.6 | 39 | 46.4 | 54.4 | 47.6 | 45.6 | - | 42.1 |
| *CfGPx3-3a* | 42.6 | 43.5 | 42.2 | 44 | 42.6 | 32.8 | 42.6 | 41.7 | 29.1 | 29.2 | 34.4 | 30 | 28.4 | - | 33.3 |
| *CfGPx3-3b* | 45.4 | 45.4 | 48.2 | 50.5 | 47.2 | 34.8 | 44.4 | 43.5 | 38.9 | 34.6 | 39.9 | 33.7 | 34.7 | - | 32.8 |
| *CfGPx3-4* | 29.9 | 30.9 | 34.5 | 42 | 41.3 | 30.9 | 30.3 | 30 | 26.6 | 34.4 | 37.5 | 30 | 30.8 | - | 30.5 |
| *CfGPx4* | 61 | 60.2 | 65.8 | - | 63.3 | 65.8 | 60.2 | 60.2 | 33.1 | 32.5 | 33.6 | 29 | 28.5 | - | 33.6 |
| *CfGPx7* | 51.9 | 52.4 | 51.8 | 50.3 | 53.1 | 53.1 | - | 51.8 | 26.8 | 31 | 30.7 | 30.7 | 29.5 | 57.1 | 27.1 |
| *PyGPx1* | 73.1 | 58.6 | 55 | 61.4 | 57.1 | 56.4 | 57.9 | 56.4 | 63.4 | 39.3 | 31.7 | 38.7 | 41.5 | - | 71.8 |
| *PyGPx3-1* | 52.5 | 50 | 46.2 | 50.8 | 47.5 | 49.2 | 48 | 48.1 | 40.3 | 48.9 | 43.1 | 41.7 | 47 | - | 43.2 |
| *PyGPx3-2* | 46.6 | 47.3 | 46.2 | 45.3 | 44.3 | 45.5 | 47.3 | 47.3 | 39.5 | 46.4 | 54.2 | 43.3 | 46.9 | - | 42.1 |
| *PyGPx3-3* | 41.7 | 39.1 | 43.5 | 44.3 | 40.6 | 44.6 | 38.2 | 40.6 | 28.7 | 40.2 | 51 | 42.3 | 42.2 | - | 36.5 |
| *PyGPx3-4* | 36.3 | 37.3 | 45.3 | 43.3 | 41.5 | 44.2 | 36.3 | 38.7 | 28.7 | 48.6 | 48.1 | 42.2 | 44.7 | - | 39.3 |
| *PyGPx3-5* | 47.2 | 46 | 48.4 | 43.7 | 44.4 | 47.5 | 41 | 47.6 | 41.6 | 40.1 | 42 | 36.3 | 39.7 | - | 40 |
| *PyGPx4* | 42.5 | 40.6 | 52.2 | - | 42.5 | 54.4 | 40.6 | 40.9 | 27.4 | 31 | 31.9 | 29.6 | 27.8 | - | 29.9 |
| *PyGPx7* | 55.4 | 55.8 | 52.2 | 49.7 | 54.3 | 52.2 | - | 51.2 | 26.1 | 31.4 | 32.1 | 29.7 | 32.1 | 54.7 | 27.8 |

Species abbreviations: Hs, Homo sapiens; Mm, Mus musculus; Xt, Xenopus tropicalis; Bt, Bos taurus; Rn, Rattus norvegicus; Xl, Xenopus laevis; Dr, Danio rerio; Gg, Gallus gallus; mollusks species: Pf, Pinctada fucata; Ut, Unio tumidus; Hdh, Haliotis discus hannai; Dp, Dreissena polymorpha; Rp, Ruditapes philippinarum; Cg, Crassostrea gigas; and Mg, Mytilus galloprovincialis.
